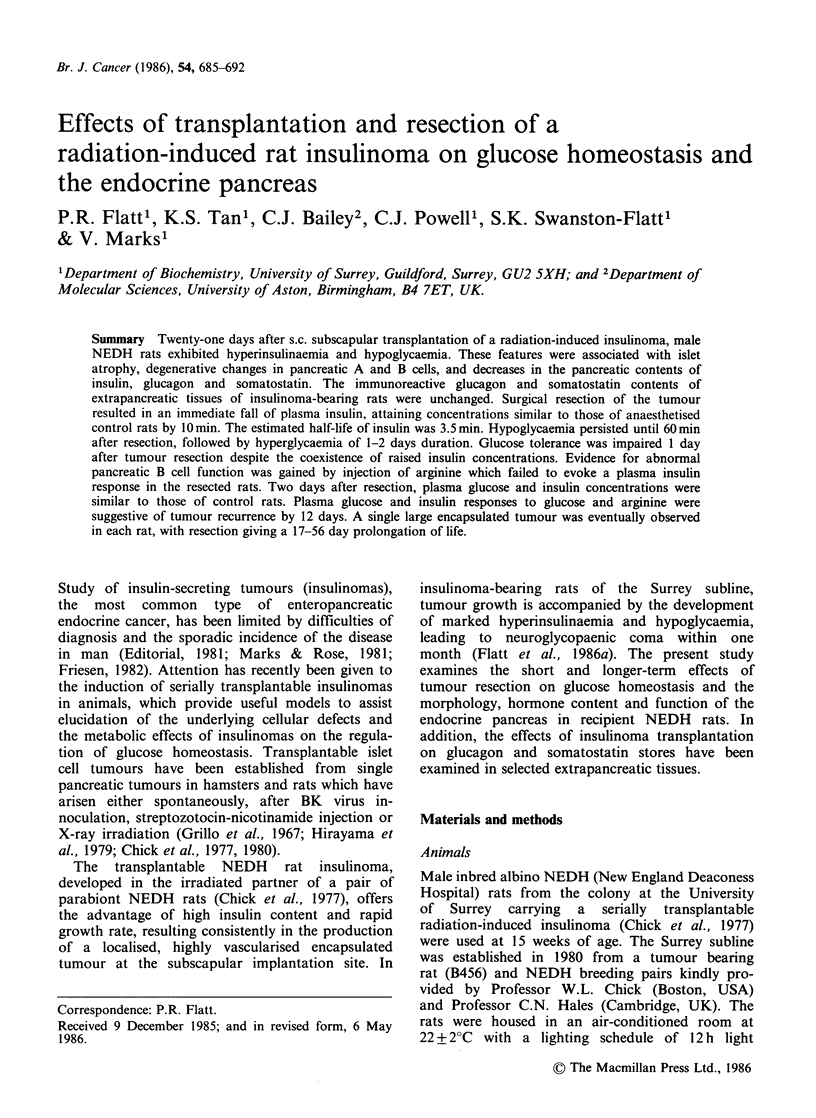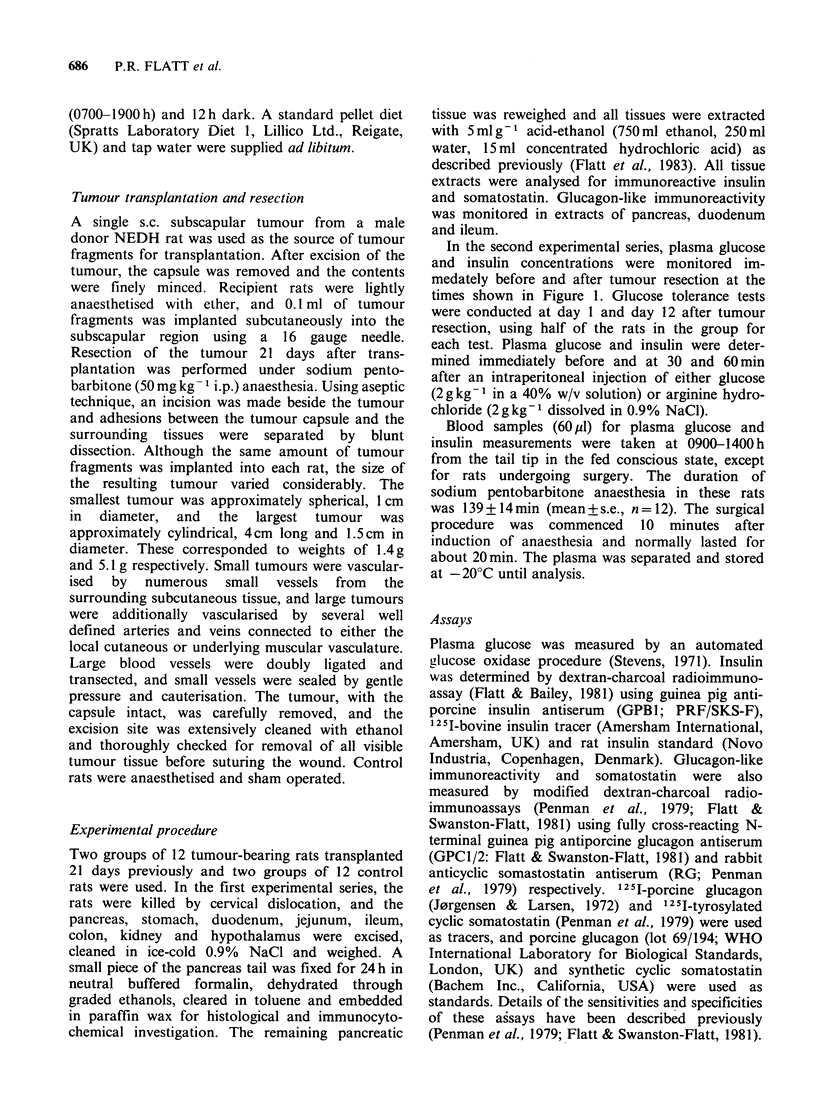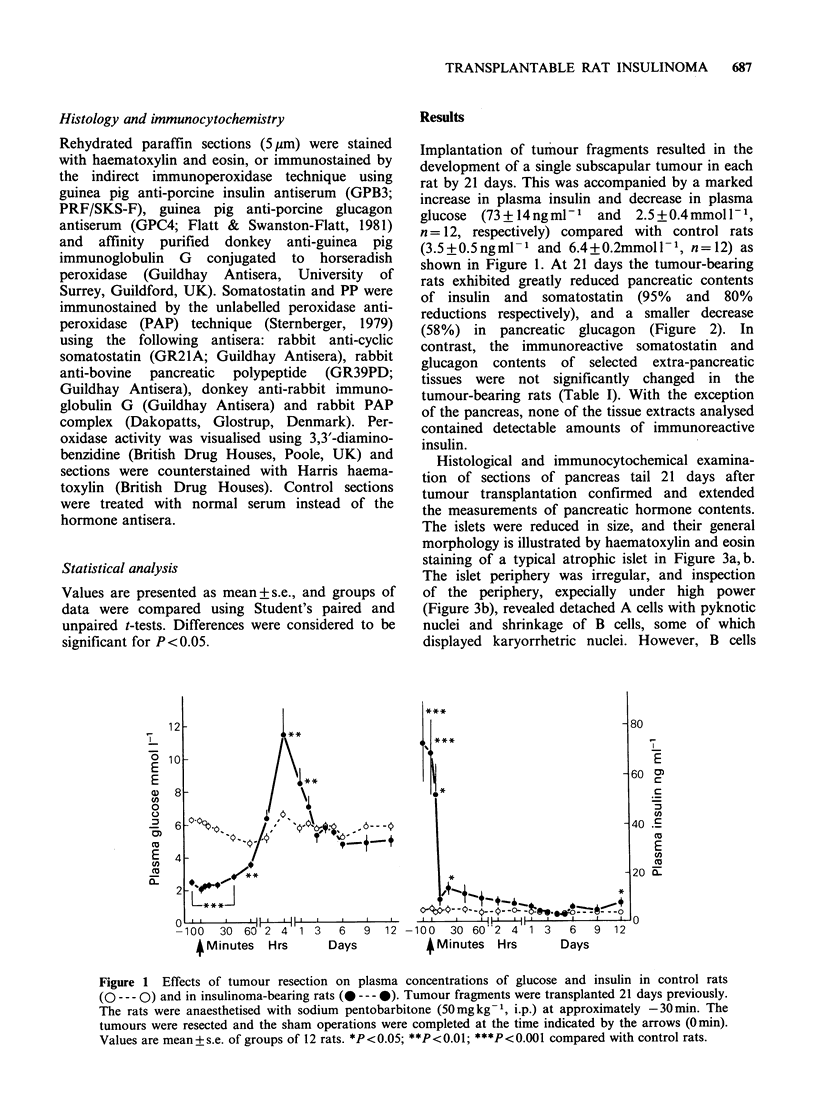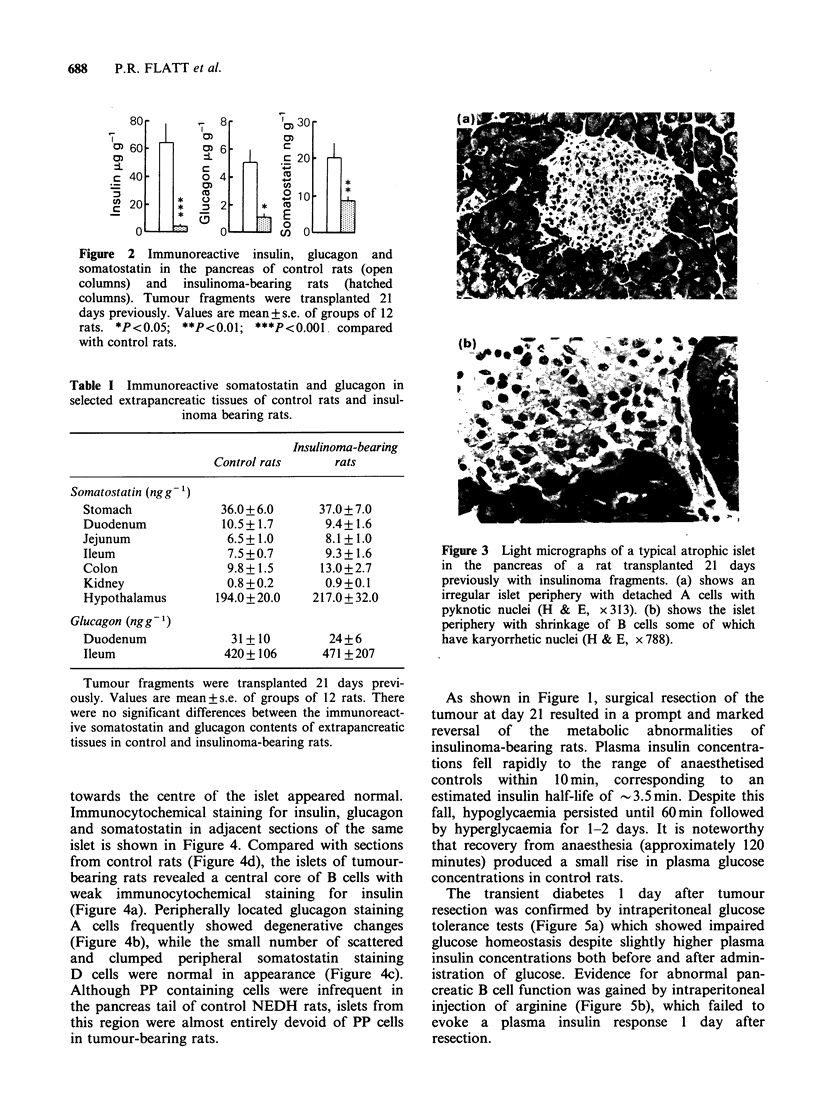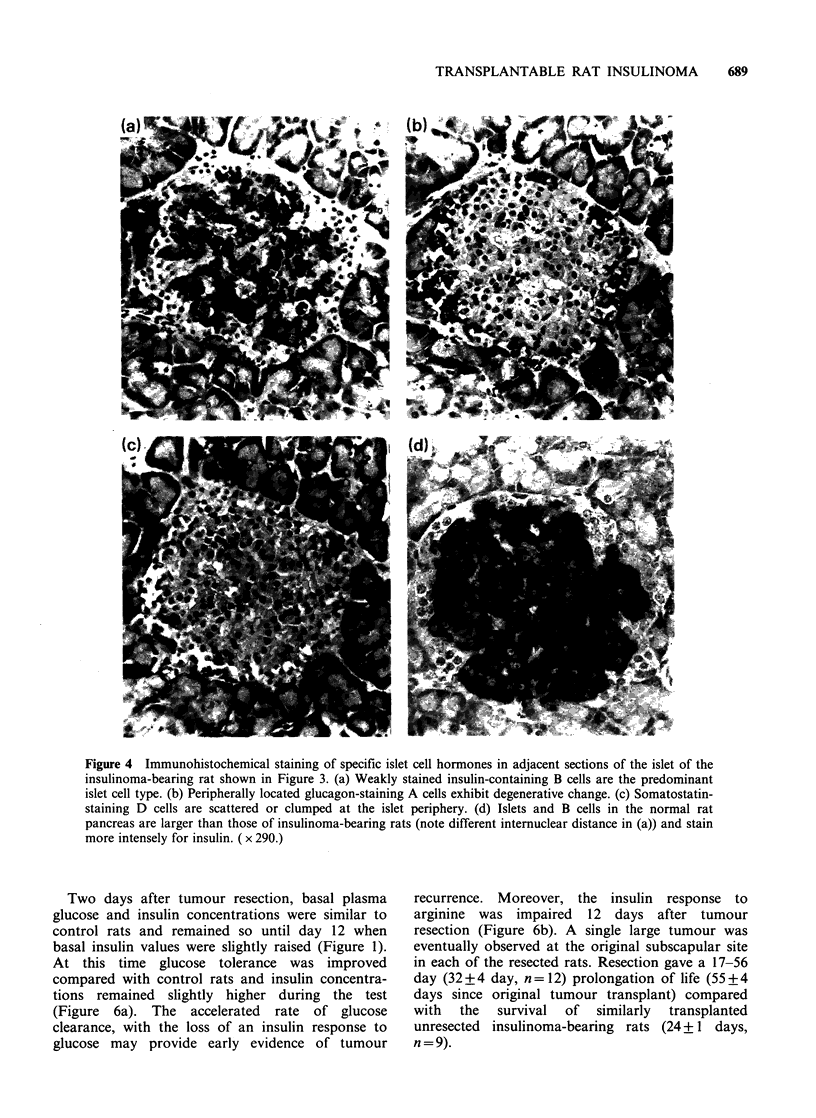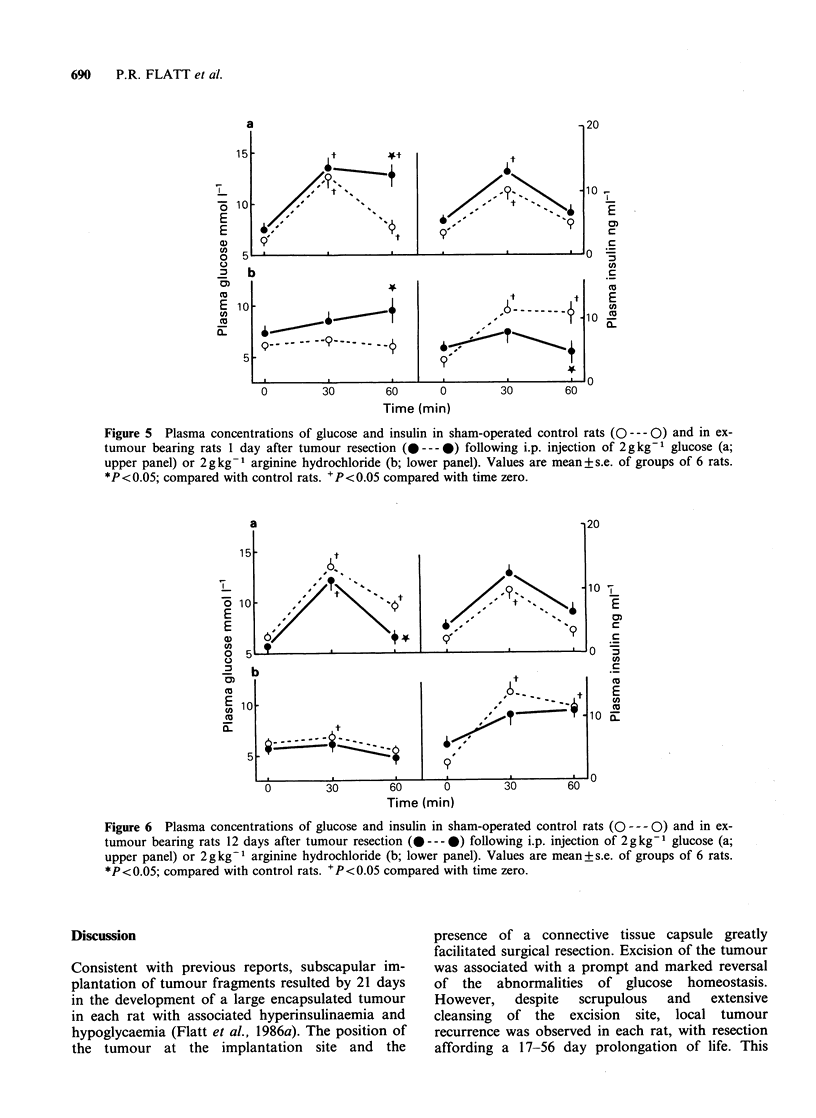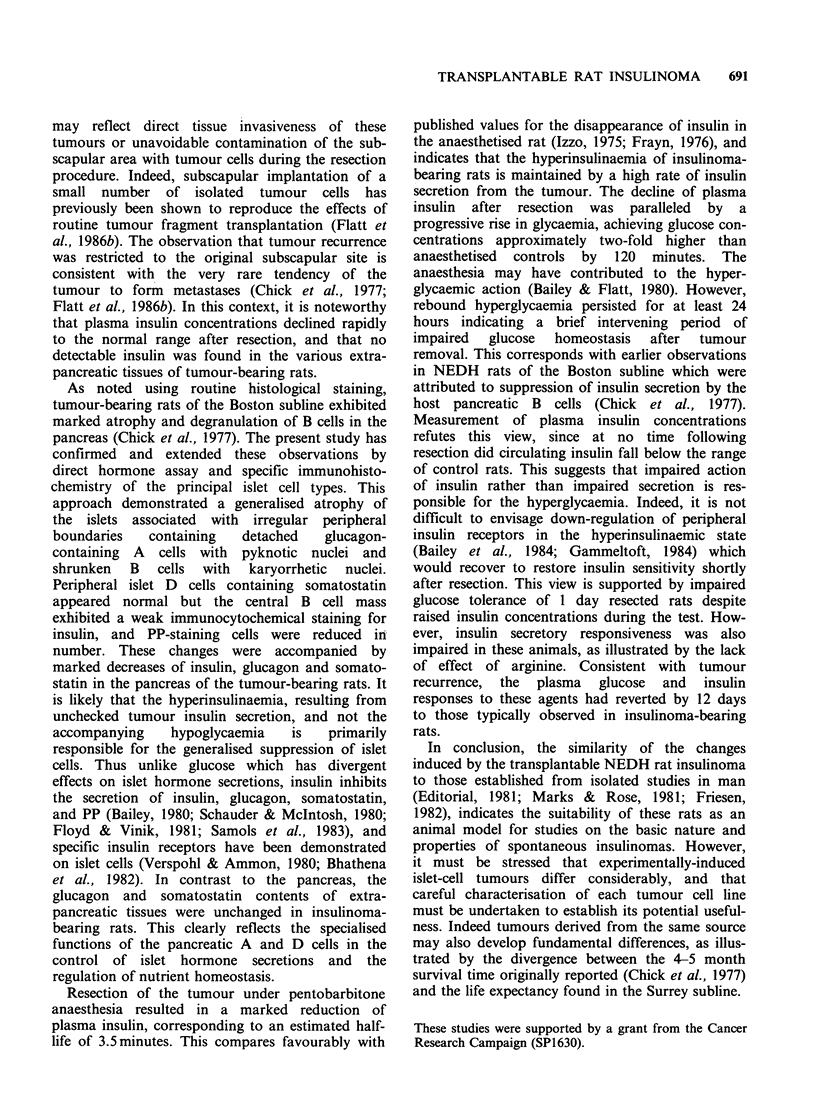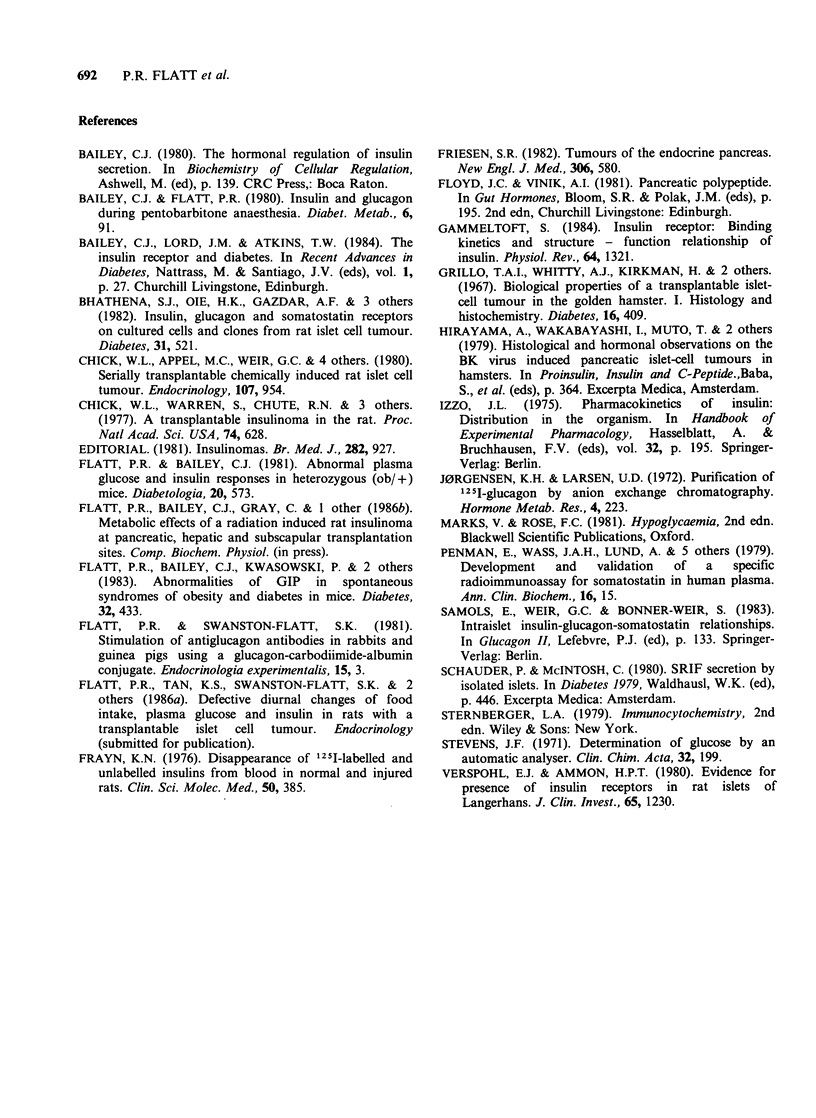# Effects of transplantation and resection of a radiation-induced rat insulinoma on glucose homeostasis and the endocrine pancreas.

**DOI:** 10.1038/bjc.1986.227

**Published:** 1986-10

**Authors:** P. R. Flatt, K. S. Tan, C. J. Bailey, C. J. Powell, S. K. Swanston-Flatt, V. Marks

## Abstract

**Images:**


*Br. J. Cancer* (1986), **54**, 685–692

### Effects of transplantation and resection of a radiation-induced rat insulinoma on glucose homeostasis and the endocrine pancreas

P.R. Flatt[1], K.S. Tan[1], C.J. Bailey[2], C.J. Powell[1], S.K. Swanston-Flatt[1] & V. Marks[1]

[1]*Department of Biochemistry, University of Surrey, Guildford, Surrey, GU2 5XH; and* [2]*Department of Molecular Sciences, University of Aston, Birmingham, B4 7ET, UK.*

**Summary** Twenty-one days after s.c. subscapular transplantation of a radiation-induced insulinoma, male NEDH rats exhibited hyperinsulinaemia and hypoglycaemia. These features were associated with islet atrophy, degenerative changes in pancreatic A and B cells, and decreases in the pancreatic contents of insulin, glucagon and somatostatin. The immunoreactive glucagon and somatostatin contents of extrapancreatic tissues of insulinoma-bearing rats were unchanged. Surgical resection of the tumour resulted in an immediate fall of plasma insulin, attaining concentrations similar to those of anaesthetised control rats by 10 min. The estimated half-life of insulin was 3.5 min. Hypoglycaemia persisted until 60 min after resection, followed by hyperglycaemia of 1–2 days duration. Glucose tolerance was impaired 1 day after tumour resection despite the coexistence of raised insulin concentrations. Evidence for abnormal pancreatic B cell function was gained by injection of arginine which failed to evoke a plasma insulin response in the resected rats. Two days after resection, plasma glucose and insulin concentrations were similar to those of control rats. Plasma glucose and insulin responses to glucose and arginine were suggestive of tumour recurrence by 12 days. A single large encapsulated tumour was eventually observed in each rat, with resection giving a 17–56 day prolongation of life.

Study of insulin-secreting tumours (insulinomas), the most common type of enteropancreatic endocrine cancer, has been limited by difficulties of diagnosis and the sporadic incidence of the disease in man (Editorial, 1981; Marks & Rose, 1981; Friesen, 1982). Attention has recently been given to the induction of serially transplantable insulinomas in animals, which provide useful models to assist elucidation of the underlying cellular defects and the metabolic effects of insulinomas on the regulation of glucose homeostasis. Transplantable islet cell tumours have been established from single pancreatic tumours in hamsters and rats which have arisen either spontaneously, after BK virus innoculation, streptozotocin-nicotinamide injection or X-ray irradiation (Grillo *et al.*, 1967; Hirayama *et al.*, 1979; Chick *et al.*, 1977, 1980).

The transplantable NEDH rat insulinoma, developed in the irradiated partner of a pair of parabiont NEDH rats (Chick *et al.*, 1977), offers the advantage of high insulin content and rapid growth rate, resulting consistently in the production of a localised, highly vascularised encapsulated tumour at the subscapular implantation site. In insulinoma-bearing rats of the Surrey subline, tumour growth is accompanied by the development of marked hyperinsulinaemia and hypoglycaemia, leading to neuroglycopaenic coma within one month (Flatt *et al.*, 1986*a*). The present study examines the short and longer-term effects of tumour resection on glucose homeostasis and the morphology, hormone content and function of the endocrine pancreas in recipient NEDH rats. In addition, the effects of insulinoma transplantation on glucagon and somatostatin stores have been examined in selected extrapancreatic tissues.

### Materials and methods

### *Animals*

Male inbred albino NEDH (New England Deaconess Hospital) rats from the colony at the University of Surrey carrying a serially transplantable radiation-induced insulinoma (Chick *et al.*, 1977) were used at 15 weeks of age. The Surrey subline was established in 1980 from a tumour bearing rat (B456) and NEDH breeding pairs kindly provided by Professor W.L. Chick (Boston, USA) and Professor C.N. Hales (Cambridge, UK). The rats were housed in an air-conditioned room at 22±2°C with a lighting schedule of 12 h light

Correspondence: P.R. Flatt.

Received 9 December 1985; and in revised form, 6 May 1986.

© The Macmillan Press Ltd., 1986

(0700–1900 h) and 12 h dark. A standard pellet diet (Spratts Laboratory Diet 1, Lillico Ltd., Reigate, UK) and tap water were supplied *ad libitum*.

*Tumour transplantation and resection*

A single s.c. subscapular tumour from a male donor NEDH rat was used as the source of tumour fragments for transplantation. After excision of the tumour, the capsule was removed and the contents were finely minced. Recipient rats were lightly anaesthetised with ether, and 0.1 ml of tumour fragments was implanted subcutaneously into the subscapular region using a 16 gauge needle. Resection of the tumour 21 days after transplantation was performed under sodium pentobarbitone (50 mg kg$^{-1}$ i.p.) anaesthesia. Using aseptic technique, an incision was made beside the tumour and adhesions between the tumour capsule and the surrounding tissues were separated by blunt dissection. Although the same amount of tumour fragments was implanted into each rat, the size of the resulting tumour varied considerably. The smallest tumour was approximately spherical, 1 cm in diameter, and the largest tumour was approximately cylindrical, 4 cm long and 1.5 cm in diameter. These corresponded to weights of 1.4 g and 5.1 g respectively. Small tumours were vascularised by numerous small vessels from the surrounding subcutaneous tissue, and large tumours were additionally vascularised by several well defined arteries and veins connected to either the local cutaneous or underlying muscular vasculature. Large blood vessels were doubly ligated and transected, and small vessels were sealed by gentle pressure and cauterisation. The tumour, with the capsule intact, was carefully removed, and the excision site was extensively cleaned with ethanol and thoroughly checked for removal of all visible tumour tissue before suturing the wound. Control rats were anaesthetised and sham operated.

*Experimental procedure*

Two groups of 12 tumour-bearing rats transplanted 21 days previously and two groups of 12 control rats were used. In the first experimental series, the rats were killed by cervical dislocation, and the pancreas, stomach, duodenum, jejunum, ileum, colon, kidney and hypothalamus were excised, cleaned in ice-cold 0.9% NaCl and weighed. A small piece of the pancreas tail was fixed for 24 h in neutral buffered formalin, dehydrated through graded ethanols, cleared in toluene and embedded in paraffin wax for histological and immunocytochemical investigation. The remaining pancreatic tissue was reweighed and all tissues were extracted with 5 ml g$^{-1}$ acid-ethanol (750 ml ethanol, 250 ml water, 15 ml concentrated hydrochloric acid) as described previously (Flatt *et al.*, 1983). All tissue extracts were analysed for immunoreactive insulin and somatostatin. Glucagon-like immunoreactivity was monitored in extracts of pancreas, duodenum and ileum.

In the second experimental series, plasma glucose and insulin concentrations were monitored immedately before and after tumour resection at the times shown in Figure 1. Glucose tolerance tests were conducted at day 1 and day 12 after tumour resection, using half of the rats in the group for each test. Plasma glucose and insulin were determined immediately before and at 30 and 60 min after an intraperitoneal injection of either glucose (2 g kg$^{-1}$ in a 40% w/v solution) or arginine hydrochloride (2 g kg$^{-1}$ dissolved in 0.9% NaCl).

Blood samples (60 $\mu$l) for plasma glucose and insulin measurements were taken at 0900–1400 h from the tail tip in the fed conscious state, except for rats undergoing surgery. The duration of sodium pentobarbitone anaesthesia in these rats was $139 \pm 14$ min (mean $\pm$ s.e., $n = 12$). The surgical procedure was commenced 10 minutes after induction of anaesthesia and normally lasted for about 20 min. The plasma was separated and stored at $-20$°C until analysis.

*Assays*

Plasma glucose was measured by an automated glucose oxidase procedure (Stevens, 1971). Insulin was determined by dextran-charcoal radioimmunoassay (Flatt & Bailey, 1981) using guinea pig antiporcine insulin antiserum (GPB1; PRF/SKS-F), $^{125}$I-bovine insulin tracer (Amersham International, Amersham, UK) and rat insulin standard (Novo Industria, Copenhagen, Denmark). Glucagon-like immunoreactivity and somatostatin were also measured by modified dextran-charcoal radioimmunoassays (Penman *et al.*, 1979; Flatt & Swanston-Flatt, 1981) using fully cross-reacting N-terminal guinea pig antiporcine glucagon antiserum (GPC1/2: Flatt & Swanston-Flatt, 1981) and rabbit anticyclic somastostatin antiserum (RG; Penman *et al.*, 1979) respectively. $^{125}$I-porcine glucagon (Jørgensen & Larsen, 1972) and $^{125}$I-tyrosylated cyclic somatostatin (Penman *et al.*, 1979) were used as tracers, and porcine glucagon (lot 69/194; WHO International Laboratory for Biological Standards, London, UK) and synthetic cyclic somatostatin (Bachem Inc., California, USA) were used as standards. Details of the sensitivities and specificities of these assays have been described previously (Penman *et al.*, 1979; Flatt & Swanston-Flatt, 1981).

*Histology and immunocytochemistry*

Rehydrated paraffin sections (5 μm) were stained with haematoxylin and eosin, or immunostained by the indirect immunoperoxidase technique using guinea pig anti-porcine insulin antiserum (GPB3; PRF/SKS-F), guinea pig anti-porcine glucagon antiserum (GPC4; Flatt & Swanston-Flatt, 1981) and affinity purified donkey anti-guinea pig immunoglobulin G conjugated to horseradish peroxidase (Guildhay Antisera, University of Surrey, Guildford, UK). Somatostatin and PP were immunostained by the unlabelled peroxidase anti-peroxidase (PAP) technique (Sternberger, 1979) using the following antisera: rabbit anti-cyclic somatostatin (GR21A; Guildhay Antisera), rabbit anti-bovine pancreatic polypeptide (GR39PD; Guildhay Antisera), donkey anti-rabbit immunoglobulin G (Guildhay Antisera) and rabbit PAP complex (Dakopatts, Glostrup, Denmark). Peroxidase activity was visualised using 3,3′-diaminobenzidine (British Drug Houses, Poole, UK) and sections were counterstained with Harris haematoxylin (British Drug Houses). Control sections were treated with normal serum instead of the hormone antisera.

*Statistical analysis*

Values are presented as mean ± s.e., and groups of data were compared using Student's paired and unpaired *t*-tests. Differences were considered to be significant for $P<0.05$.

### Results

Implantation of tumour fragments resulted in the development of a single subscapular tumour in each rat by 21 days. This was accompanied by a marked increase in plasma insulin and decrease in plasma glucose ($73\pm14\,ng\,ml^{-1}$ and $2.5\pm0.4\,mmol\,l^{-1}$, $n=12$, respectively) compared with control rats ($3.5\pm0.5\,ng\,ml^{-1}$ and $6.4\pm0.2\,mmol\,l^{-1}$, $n=12$) as shown in Figure 1. At 21 days the tumour-bearing rats exhibited greatly reduced pancreatic contents of insulin and somatostatin (95% and 80% reductions respectively), and a smaller decrease (58%) in pancreatic glucagon (Figure 2). In contrast, the immunoreactive somatostatin and glucagon contents of selected extra-pancreatic tissues were not significantly changed in the tumour-bearing rats (Table I). With the exception of the pancreas, none of the tissue extracts analysed contained detectable amounts of immunoreactive insulin.

Histological and immunocytochemical examination of sections of pancreas tail 21 days after tumour transplantation confirmed and extended the measurements of pancreatic hormone contents. The islets were reduced in size, and their general morphology is illustrated by haematoxylin and eosin staining of a typical atrophic islet in Figure 3a, b. The islet periphery was irregular, and inspection of the periphery, expecially under high power (Figure 3b), revealed detached A cells with pyknotic nuclei and shrinkage of B cells, some of which displayed karyorrhetic nuclei. However, B cells

**Figure 1** Effects of tumour resection on plasma concentrations of glucose and insulin in control rats (○---○) and in insulinoma-bearing rats (●---●). Tumour fragments were transplanted 21 days previously. The rats were anaesthetised with sodium pentobarbitone ($50\,mg\,kg^{-1}$, i.p.) at approximately −30 min. The tumours were resected and the sham operations were completed at the time indicated by the arrows (0 min). Values are mean ± s.e. of groups of 12 rats. *$P<0.05$; **$P<0.01$; ***$P<0.001$ compared with control rats.

**Figure 2** Immunoreactive insulin, glucagon and somatostatin in the pancreas of control rats (open columns) and insulinoma-bearing rats (hatched columns). Tumour fragments were transplanted 21 days previously. Values are mean ± s.e. of groups of 12 rats. $^{*}P<0.05$; $^{**}P<0.01$; $^{***}P<0.001$, compared with control rats.

**Table I** Immunoreactive somatostatin and glucagon in selected extrapancreatic tissues of control rats and insulinoma bearing rats.

| | *Control rats* | *Insulinoma-bearing rats* |
|---|---|---|
| *Somatostatin* ($ng\,g^{-1}$) | | |
| Stomach | 36.0 ± 6.0 | 37.0 ± 7.0 |
| Duodenum | 10.5 ± 1.7 | 9.4 ± 1.6 |
| Jejunum | 6.5 ± 1.0 | 8.1 ± 1.0 |
| Ileum | 7.5 ± 0.7 | 9.3 ± 1.6 |
| Colon | 9.8 ± 1.5 | 13.0 ± 2.7 |
| Kidney | 0.8 ± 0.2 | 0.9 ± 0.1 |
| Hypothalamus | 194.0 ± 20.0 | 217.0 ± 32.0 |
| *Glucagon* ($ng\,g^{-1}$) | | |
| Duodenum | 31 ± 10 | 24 ± 6 |
| Ileum | 420 ± 106 | 471 ± 207 |

Tumour fragments were transplanted 21 days previously. Values are mean ± s.e. of groups of 12 rats. There were no significant differences between the immunoreactive somatostatin and glucagon contents of extrapancreatic tissues in control and insulinoma-bearing rats.

towards the centre of the islet appeared normal. Immunocytochemical staining for insulin, glucagon and somatostatin in adjacent sections of the same islet is shown in Figure 4. Compared with sections from control rats (Figure 4d), the islets of tumour-bearing rats revealed a central core of B cells with weak immunocytochemical staining for insulin (Figure 4a). Peripherally located glucagon staining A cells frequently showed degenerative changes (Figure 4b), while the small number of scattered and clumped peripheral somatostatin staining D cells were normal in appearance (Figure 4c). Although PP containing cells were infrequent in the pancreas tail of control NEDH rats, islets from this region were almost entirely devoid of PP cells in tumour-bearing rats.

**Figure 3** Light micrographs of a typical atrophic islet in the pancreas of a rat transplanted 21 days previously with insulinoma fragments. (a) shows an irregular islet periphery with detached A cells with pyknotic nuclei (H & E, ×313). (b) shows the islet periphery with shrinkage of B cells some of which have karyorrhetic nuclei (H & E, ×788).

As shown in Figure 1, surgical resection of the tumour at day 21 resulted in a prompt and marked reversal of the metabolic abnormalities of insulinoma-bearing rats. Plasma insulin concentrations fell rapidly to the range of anaesthetised controls within 10 min, corresponding to an estimated insulin half-life of ~3.5 min. Despite this fall, hypoglycaemia persisted until 60 min followed by hyperglycaemia for 1–2 days. It is noteworthy that recovery from anaesthesia (approximately 120 minutes) produced a small rise in plasma glucose concentrations in control rats.

The transient diabetes 1 day after tumour resection was confirmed by intraperitoneal glucose tolerance tests (Figure 5a) which showed impaired glucose homeostasis despite slightly higher plasma insulin concentrations both before and after administration of glucose. Evidence for abnormal pancreatic B cell function was gained by intraperitoneal injection of arginine (Figure 5b), which failed to evoke a plasma insulin response 1 day after resection.

**Figure 4** Immunohistochemical staining of specific islet cell hormones in adjacent sections of the islet of the insulinoma-bearing rat shown in Figure 3. (a) Weakly stained insulin-containing B cells are the predominant islet cell type. (b) Peripherally located glucagon-staining A cells exhibit degenerative change. (c) Somatostatin-staining D cells are scattered or clumped at the islet periphery. (d) Islets and B cells in the normal rat pancreas are larger than those of insulinoma-bearing rats (note different internuclear distance in (a)) and stain more intensely for insulin. ($\times 290$.)

Two days after tumour resection, basal plasma glucose and insulin concentrations were similar to control rats and remained so until day 12 when basal insulin values were slightly raised (Figure 1). At this time glucose tolerance was improved compared with control rats and insulin concentrations remained slightly higher during the test (Figure 6a). The accelerated rate of glucose clearance, with the loss of an insulin response to glucose may provide early evidence of tumour recurrence. Moreover, the insulin response to arginine was impaired 12 days after tumour resection (Figure 6b). A single large tumour was eventually observed at the original subscapular site in each of the resected rats. Resection gave a 17–56 day ($32 \pm 4$ day, $n = 12$) prolongation of life ($55 \pm 4$ days since original tumour transplant) compared with the survival of similarly transplanted unresected insulinoma-bearing rats ($24 \pm 1$ days, $n = 9$).

**Figure 5** Plasma concentrations of glucose and insulin in sham-operated control rats (○---○) and in ex-tumour bearing rats 1 day after tumour resection (●---●) following i.p. injection of $2\,g\,kg^{-1}$ glucose (a; upper panel) or $2\,g\,kg^{-1}$ arginine hydrochloride (b; lower panel). Values are mean±s.e. of groups of 6 rats. $^{*}P<0.05$; compared with control rats. $^{+}P<0.05$ compared with time zero.

**Figure 6** Plasma concentrations of glucose and insulin in sham-operated control rats (○---○) and in ex-tumour bearing rats 12 days after tumour resection (●---●) following i.p. injection of $2\,g\,kg^{-1}$ glucose (a; upper panel) or $2\,g\,kg^{-1}$ arginine hydrochloride (b; lower panel). Values are mean±s.e. of groups of 6 rats. $^{*}P<0.05$; compared with control rats. $^{+}P<0.05$ compared with time zero.

### Discussion

Consistent with previous reports, subscapular implantation of tumour fragments resulted by 21 days in the development of a large encapsulated tumour in each rat with associated hyperinsulinaemia and hypoglycaemia (Flatt *et al.*, 1986*a*). The position of the tumour at the implantation site and the presence of a connective tissue capsule greatly facilitated surgical resection. Excision of the tumour was associated with a prompt and marked reversal of the abnormalities of glucose homeostasis. However, despite scrupulous and extensive cleansing of the excision site, local tumour recurrence was observed in each rat, with resection affording a 17–56 day prolongation of life. This may reflect direct tissue invasiveness of these tumours or unavoidable contamination of the subscapular area with tumour cells during the resection procedure. Indeed, subscapular implantation of a small number of isolated tumour cells has previously been shown to reproduce the effects of routine tumour fragment transplantation (Flatt *et al.*, 1986*b*). The observation that tumour recurrence was restricted to the original subscapular site is consistent with the very rare tendency of the tumour to form metastases (Chick *et al.*, 1977; Flatt *et al.*, 1986*b*). In this context, it is noteworthy that plasma insulin concentrations declined rapidly to the normal range after resection, and that no detectable insulin was found in the various extra-pancreatic tissues of tumour-bearing rats.

As noted using routine histological staining, tumour-bearing rats of the Boston subline exhibited marked atrophy and degranulation of B cells in the pancreas (Chick *et al.*, 1977). The present study has confirmed and extended these observations by direct hormone assay and specific immunohistochemistry of the principal islet cell types. This approach demonstrated a generalised atrophy of the islets associated with irregular peripheral boundaries containing detached glucagon-containing A cells with pyknotic nuclei and shrunken B cells with karyorrhetic nuclei. Peripheral islet D cells containing somatostatin appeared normal but the central B cell mass exhibited a weak immunocytochemical staining for insulin, and PP-staining cells were reduced in number. These changes were accompanied by marked decreases of insulin, glucagon and somatostatin in the pancreas of the tumour-bearing rats. It is likely that the hyperinsulinaemia, resulting from unchecked tumour insulin secretion, and not the accompanying hypoglycaemia is primarily responsible for the generalised suppression of islet cells. Thus unlike glucose which has divergent effects on islet hormone secretions, insulin inhibits the secretion of insulin, glucagon, somatostatin, and PP (Bailey, 1980; Schauder & McIntosh, 1980; Floyd & Vinik, 1981; Samols *et al.*, 1983), and specific insulin receptors have been demonstrated on islet cells (Verspohl & Ammon, 1980; Bhathena *et al.*, 1982). In contrast to the pancreas, the glucagon and somatostatin contents of extra-pancreatic tissues were unchanged in insulinoma-bearing rats. This clearly reflects the specialised functions of the pancreatic A and D cells in the control of islet hormone secretions and the regulation of nutrient homeostasis.

Resection of the tumour under pentobarbitone anaesthesia resulted in a marked reduction of plasma insulin, corresponding to an estimated half-life of 3.5 minutes. This compares favourably with published values for the disappearance of insulin in the anaesthetised rat (Izzo, 1975; Frayn, 1976), and indicates that the hyperinsulinaemia of insulinoma-bearing rats is maintained by a high rate of insulin secretion from the tumour. The decline of plasma insulin after resection was paralleled by a progressive rise in glycaemia, achieving glucose concentrations approximately two-fold higher than anaesthetised controls by 120 minutes. The anaesthesia may have contributed to the hyperglycaemic action (Bailey & Flatt, 1980). However, rebound hyperglycaemia persisted for at least 24 hours indicating a brief intervening period of impaired glucose homeostasis after tumour removal. This corresponds with earlier observations in NEDH rats of the Boston subline which were attributed to suppression of insulin secretion by the host pancreatic B cells (Chick *et al.*, 1977). Measurement of plasma insulin concentrations refutes this view, since at no time following resection did circulating insulin fall below the range of control rats. This suggests that impaired action of insulin rather than impaired secretion is responsible for the hyperglycaemia. Indeed, it is not difficult to envisage down-regulation of peripheral insulin receptors in the hyperinsulinaemic state (Bailey *et al.*, 1984; Gammeltoft, 1984) which would recover to restore insulin sensitivity shortly after resection. This view is supported by impaired glucose tolerance of 1 day resected rats despite raised insulin concentrations during the test. However, insulin secretory responsiveness was also impaired in these animals, as illustrated by the lack of effect of arginine. Consistent with tumour recurrence, the plasma glucose and insulin responses to these agents had reverted by 12 days to those typically observed in insulinoma-bearing rats.

In conclusion, the similarity of the changes induced by the transplantable NEDH rat insulinoma to those established from isolated studies in man (Editorial, 1981; Marks & Rose, 1981; Friesen, 1982), indicates the suitability of these rats as an animal model for studies on the basic nature and properties of spontaneous insulinomas. However, it must be stressed that experimentally-induced islet-cell tumours differ considerably, and that careful characterisation of each tumour cell line must be undertaken to establish its potential usefulness. Indeed tumours derived from the same source may also develop fundamental differences, as illustrated by the divergence between the 4–5 month survival time originally reported (Chick *et al.*, 1977) and the life expectancy found in the Surrey subline.

These studies were supported by a grant from the Cancer Research Campaign (SP1630).

### References

BAILEY, C.J. (1980). The hormonal regulation of insulin secretion. In *Biochemistry of Cellular Regulation*, Ashwell, M. (ed), p. 139. CRC Press,: Boca Raton.

BAILEY, C.J. & FLATT, P.R. (1980). Insulin and glucagon during pentobarbitone anaesthesia. *Diabet. Metab.*, **6**, 91.

BAILEY, C.J., LORD, J.M. & ATKINS, T.W. (1984). The insulin receptor and diabetes. In *Recent Advances in Diabetes*, Nattrass, M. & Santiago, J.V. (eds), vol. **1**, p. 27. Churchill Livingstone, Edinburgh.

BHATHENA, S.J., OIE, H.K., GAZDAR, A.F. & 3 others (1982). Insulin, glucagon and somatostatin receptors on cultured cells and clones from rat islet cell tumour. *Diabetes*, **31**, 521.

CHICK, W.L., APPEL, M.C., WEIR, G.C. & 4 others. (1980). Serially transplantable chemically induced rat islet cell tumour. *Endocrinology*, **107**, 954.

CHICK, W.L., WARREN, S., CHUTE, R.N. & 3 others. (1977). A transplantable insulinoma in the rat. *Proc. Natl Acad. Sci. USA*, **74**, 628.

EDITORIAL. (1981). Insulinomas. *Br. Med. J.*, **282**, 927.

FLATT, P.R. & BAILEY, C.J. (1981). Abnormal plasma glucose and insulin responses in heterozygous (ob/+) mice. *Diabetologia*, **20**, 573.

FLATT, P.R., BAILEY, C.J., GRAY, C. & 1 other (1986*b*). Metabolic effects of a radiation induced rat insulinoma at pancreatic, hepatic and subscapular transplantation sites. *Comp. Biochem. Physiol.* (in press).

FLATT, P.R., BAILEY, C.J., KWASOWSKI, P. & 2 others (1983). Abnormalities of GIP in spontaneous syndromes of obesity and diabetes in mice. *Diabetes*, **32**, 433.

FLATT, P.R. & SWANSTON-FLATT, S.K. (1981). Stimulation of antiglucagon antibodies in rabbits and guinea pigs using a glucagon-carbodiimide-albumin conjugate. *Endocrinologia experimentalis*, **15**, 3.

FLATT, P.R., TAN, K.S., SWANSTON-FLATT, S.K. & 2 others (1986*a*). Defective diurnal changes of food intake, plasma glucose and insulin in rats with a transplantable islet cell tumour. *Endocrinology* (submitted for publication).

FRAYN, K.N. (1976). Disappearance of $^{125}$I-labelled and unlabelled insulins from blood in normal and injured rats. *Clin. Sci. Molec. Med.*, **50**, 385.

FRIESEN, S.R. (1982). Tumours of the endocrine pancreas. *New Engl. J. Med.*, **306**, 580.

FLOYD, J.C. & VINIK, A.I. (1981). Pancreatic polypeptide. In *Gut Hormones*, Bloom, S.R. & Polak, J.M. (eds), p. 195. 2nd edn, Churchill Livingstone: Edinburgh.

GAMMELTOFT, S. (1984). Insulin receptor: Binding kinetics and structure – function relationship of insulin. *Physiol. Rev.*, **64**, 1321.

GRILLO, T.A.I., WHITTY, A.J., KIRKMAN, H. & 2 others. (1967). Biological properties of a transplantable islet-cell tumour in the golden hamster. I. Histology and histochemistry. *Diabetes*, **16**, 409.

HIRAYAMA, A., WAKABAYASHI, I., MUTO, T. & 2 others (1979). Histological and hormonal observations on the BK virus induced pancreatic islet-cell tumours in hamsters. In *Proinsulin, Insulin and C-Peptide.*,Baba, S., *et al.* (eds), p. 364. Excerpta Medica, Amsterdam.

IZZO, J.L. (1975). Pharmacokinetics of insulin: Distribution in the organism. In *Handbook of Experimental Pharmacology*, Hasselblatt, A. & Bruchhausen, F.V. (eds), vol. **32**, p. 195. Springer-Verlag: Berlin.

JØRGENSEN, K.H. & LARSEN, U.D. (1972). Purification of $^{125}$I-glucagon by anion exchange chromatography. *Hormone Metab. Res.*, **4**, 223.

MARKS, V. & ROSE, F.C. (1981). *Hypoglycaemia*, 2nd edn. Blackwell Scientific Publications, Oxford.

PENMAN, E., WASS, J.A.H., LUND, A. & 5 others (1979). Development and validation of a specific radioimmunoassay for somatostatin in human plasma. *Ann. Clin. Biochem.*, **16**, 15.

SAMOLS, E., WEIR, G.C. & BONNER-WEIR, S. (1983). Intraislet insulin-glucagon-somatostatin relationships. In *Glucagon II*, Lefebvre, P.J. (ed), p. 133. Springer-Verlag: Berlin.

SCHAUDER, P. & McINTOSH, C. (1980). SRIF secretion by isolated islets. In *Diabetes 1979*, Waldhausl, W.K. (ed), p. 446. Excerpta Medica: Amsterdam.

STERNBERGER, L.A. (1979). *Immunocytochemistry*, 2nd edn. Wiley & Sons: New York.

STEVENS, J.F. (1971). Determination of glucose by an automatic analyser. *Clin. Chim. Acta*, **32**, 199.

VERSPOHL, E.J. & AMMON, H.P.T. (1980). Evidence for presence of insulin receptors in rat islets of Langerhans. *J. Clin. Invest.*, **65**, 1230.